# Mitrofanovite Pt_3_Te_4_: A Topological Metal with Termination-Dependent Surface Band Structure and Strong Spin Polarization

**DOI:** 10.1021/acsnano.1c04766

**Published:** 2021-09-02

**Authors:** Jun Fujii, Barun Ghosh, Ivana Vobornik, Anan Bari Sarkar, Debashis Mondal, Chia-Nung Kuo, François C. Bocquet, Lixue Zhang, Danil W. Boukhvalov, Chin Shan Lue, Amit Agarwal, Antonio Politano

**Affiliations:** † CNR-IOM, TASC Laboratory, Area Science Park-Basovizza, 34139 Trieste, Italy; ‡ Department of Physics, Indian Institute of Technology Kanpur, Kanpur 208016, India; § Department of Physics, National Cheng Kung University, 1 Ta-Hsueh Road, 70101 Tainan, Taiwan; ∥ Peter Grünberg Institut (PGI-3), Forschungszentrum Jülich, 52425 Jülich, Germany; ⊥ Jülich Aachen Research Alliance (JARA), Fundamentals of Future Information Technology, 52425 Jülich, Germany; # College of Chemistry and Chemical Engineering, Qingdao University, Qingdao 266071, Shandong, P. R. China; 7 College of Science, Institute of Materials Physics and Chemistry, Nanjing Forestry University, Nanjing 210037, P. R. China; 8 Institute of Physics and Technology, Ural Federal University, Mira Street 19, 620002 Ekaterinburg, Russia; 9 INSTM and Department of Physical and Chemical Sciences, University of L’Aquila, via Vetoio, 67100 L’Aquila (AQ), Italy; 10 CNR-IMM Istituto per la Microelettronica e Microsistemi, VIII strada 5, I-95121 Catania, Italy; ⊗ Taiwan Consortium of Emergent Crystalline Materials, Ministry of Science and Technology, Taipei 10601, Taiwan

**Keywords:** topological metals, surface states, STM/STS, spintronics, ARPES

## Abstract

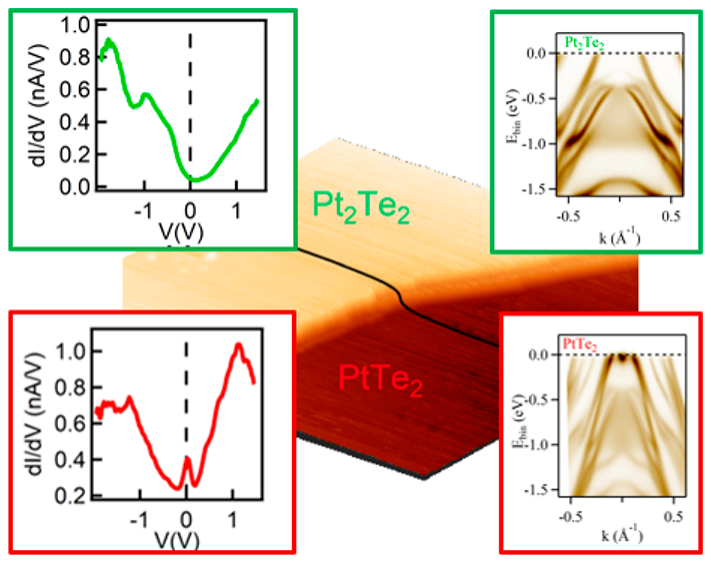

Due to their peculiar quasiparticle excitations, topological metals have high potential for applications in the fields of spintronics, catalysis, and superconductivity. Here, by combining spin- and angle-resolved photoemission spectroscopy, scanning tunneling microscopy/spectroscopy, and density functional theory, we discover surface-termination-dependent topological electronic states in the recently discovered mitrofanovite Pt_3_Te_4_. Mitrofanovite crystal is formed by alternating, van der Waals bound layers of Pt_2_Te_2_ and PtTe_2_. Our results demonstrate that mitrofanovite is a topological metal with termination-dependent (i) electronic band structure and (ii) spin texture. Despite their distinct electronic character, both surface terminations are characterized by electronic states exhibiting strong spin polarization with a node at the Γ point and sign reversal across the Γ point, indicating their topological nature and the possibility of realizing two distinct electronic configurations (both of them with topological features) on the surface of the same material.

## Introduction

Topological metals are materials with nontrivial band crossings or band inversions near the Fermi energy, giving rise to peculiar quasiparticle excitations.^1−8^ They can be classified based on the dimensionality and degeneracy of their band crossings.^9^ Prominent examples include Dirac,^10^ Weyl,^11^ nodal-line,^12^ and nodal-surface metals.^9^ In topological metals, bulk superconductivity can also coexist with topologically nontrivial states, as demonstrated for PbTaSe_2_^13^ enabling the intriguing perspective of Majorana Fermions in solid-state physics. Furthermore, the heterostructures of topological metals are being pursued as suitable candidates for potential applications in quantum computing.^14^ The fascinating technological capabilities of topological metals are also confirmed by recent reports indicating their superior efficiency in catalytic reactions and hydrogen production, as demonstrated for the case of the Pt-based alloys PtAl and PtGa.^15^

Among the various families of materials showing gapless Dirac Fermions, the transition-metal dichalcogenide TMX_2_ (TM = Pd, Pt; X = Se, Te), crystallizing in the same structure as the naturally occurring mineral “moncheite”,^16^ was demonstrated to host type-II Dirac fermions,^17^ with application capabilities in plasmonics,^10^ catalysis,^18^ nanoelectronics,^19^ and wearable electronics.^20^ These properties can be tuned by varying (i) the position of the Fermi level with respect to the degenerate Dirac (or Weyl/nodal line) point and (ii) the strength of the spin–orbit coupling.

Mitrofanovite Pt_3_Te_4_, belonging to the “moncheite” family of materials with trigonal space group *R*3̅*m* (No. 166), has recently been discovered as a natural mineral in the Kola Peninsula, Russia,^21^ and in different zones in the Canadian Shield.^22^ Its atomic structure is constituted by alternating layers of hexagonal PtTe_2_ and Pt_2_Te_2_ (or PtTe) which are stacked along the vertical direction and held together by weak van der Waals interactions. Very recently, mitrofanovite has been demonstrated to be an efficient and stable catalyst for hydrogen evolution reaction (HER) with an overpotential of 39.6 mV and a Tafel slope of 32.7 mV/dec together with a high current density exceeding 7000 mA/cm^2^.^23^

Here, we explore the electronic properties of Pt_3_Te_4_ by means of scanning tunneling microscopy (STM)/spectroscopy (STS) and spin- and angle-resolved photoemission spectroscopy (spin-ARPES) in conjugation with density functional theory (DFT). We demonstrate that mitrofanovite is a topological metal hosting spin-polarized surface states. Interestingly, we find that Pt_3_Te_4_ has two distinct surface terminations with radically different electronic properties. These distinct terminations are observed at different terraces on the same face of the cleaved crystal. Despite differences in the corresponding electronic band structure, both surface terminations host spin-polarized states, exhibiting typical polarization reversal across the zone center, characteristic of spin-momentum locking. Thus, mitrofanovite offers termination-dependent electronic and surface properties enabling tunable device functionalities for nanoelectronics, spintronics, optoelectronics, and plasmonic applications.

## Results and Discussion

### Identification of Two Distinct Surface Terminations

**Figure 1:**
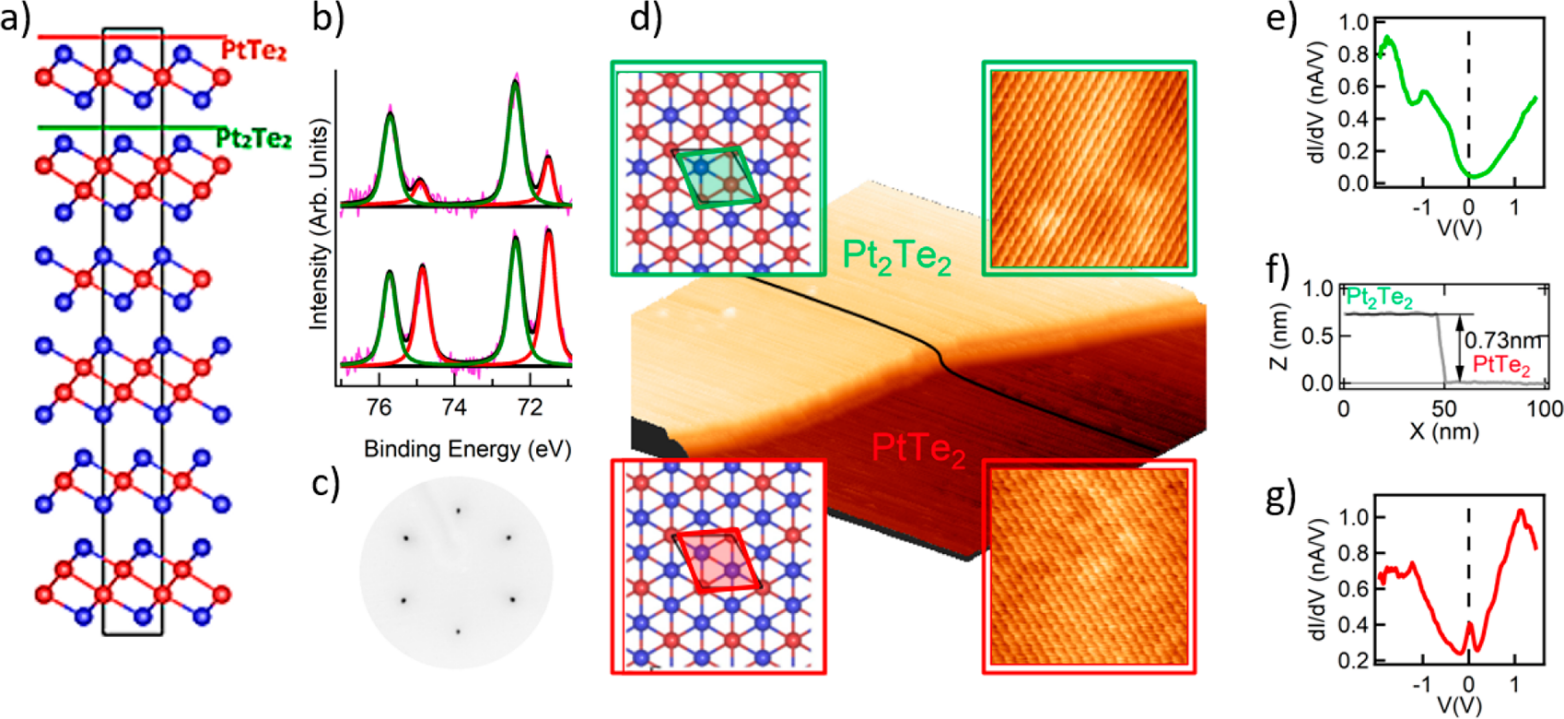
(a) Side view of the Pt_3_Te_4_ crystal structure with the two different cleavage planes—Pt_2_Te_2_ (green) and PtTe_2_ (red) terminations are identified. (b) Pt-4f XPS spectra for the Pt_2_Te_2_ (top) and PtTe_2_ (bottom) terminations, respectively. (c) Hexagonal LEED pattern obtained on both terminations. (d) 3D STM topographic image (100 nm × 100 nm) showing the adjacent terraces of different terminations separated by a step; the four insets framed green (red) are the top view of the crystal structure and the atomically resolved STM image (7 nm × 7 nm) for the Pt_2_Te_2_ (PtTe_2_) terminations. (e and g) STS spectra measured on the Pt_2_Te_2_ and the PtTe_2_ terraces. We observe a zero-bias peak only on the PtTe_2_ termination. (f) Height profile along the black line in panel d.

The unit cell of Pt_3_Te_4_ is composed of alternating blocks of PtTe_2_ and Pt_2_Te_2_ units stacked vertically, as depicted in Figure 1a for the side view and in the red and green boxes in the inset of Figure 1d for top views. Specifically, in the PtTe_2_ subunit, one atomic Pt layer is sandwiched between the two Te layers. Contrariwise, in the Pt_2_Te_2_ subunit, two layers of Pt atoms are sandwiched between two Te layers. For such a crystal structure, two cleavage planes are feasible, resulting either in (i) a PtTe_2_ termination (marked by a red line in Figure 1a) or (ii) a Pt_2_Te_2_ termination (green line in Figure 1a). For the sake of completeness, also a PtTe termination is in principle feasible. However, as the energy per surface unit for the PtTe-terminated slab is more than 1 eV larger than for the PtTe_2_- and Pt_2_Te_2_-terminated slab, this type of termination can be excluded from further discussion.

Thus, the existence of two distinct interfaces at the surface of the mitrofanovite crystal enables tunable electronic, spintronic, and plasmonic properties of the electrode contact for exploitation in micro- and nanoelectronics.

By means of high-resolution X-ray spectroscopy (XPS) on an as-cleaved sample, we identified two distinct spectral components in the region of Pt-4f core levels. In our experimental configuration (low-energy photons of 120 eV in normal emission geometry), the probing depth is 4.2 ± 0.1 Å, based on the effective attenuation length according to the Tanuma–Powel–Penn (TPP-2M) formula.^24^ This is comparable with the size of the single Pt_2_Te_2_ subunits (4.02 Å). Therefore, we expect that the two surfaces with different terminations will be reflected by different Pt binding energies (BE).

Consistent with our expectations, we observe a splitting in the Pt-4f core levels, with *J* = 7/2 components having a BE of 71.5 and 72.4 eV. To assign the two spectral components, we computed core-level shifts based on the charge distribution in the Pt_2_Te_2_ subunit. We found that, in the Pt–Te bonds, the charge on the Pt sites is reduced by 0.363 electrons compared to the Pt–Pt bonds. The intensity ratio between the two spectral components is 1.0 ± 0.1 in the Pt_2_Te_2_-termination. Concerning the PtTe_2_-terminated surface, we observe a suppression of the Pt-4f component related to Pt–Pt bonds up to 75%. The total quenching was not obtained, due to the combined effect of the limited spatial resolution (beam size 150 × 50 μm^2^) and the insufficient probing depth to selectively choose the contributions from the PtTe_2_ subunit only, whose size is 2.71 Å.

The two different surface terminations were further identified by means of scanning tunneling microscopy (STM) experiments. The three-dimensional STM topographic image (100 × 100 nm^2^) in Figure 1d highlights the adjacent large terraces separated by a step on a cleaved surface of mitrofanovite. The height profile shown in Figure 1f of the step in Figure 1d reveals a stacking of 0.73 nm, with respect to the Pt_2_Te_2_ termination. This stacking distance is consistent with the distance between the Pt_2_Te_2_ (depicted in the left green-framed inset of Figure 1d) and PtTe_2_ (depicted in the left red-framed inset of Figure 1d) subunits shown schematically in Figure 1a. The corresponding atomic resolution STM image of the Pt_2_Te_2_ (PtTe_2_) terrace is shown in the right inset of Figure 1d, within the green (red) box.

To compare the local electronic density of states (LDOS) on the two distinct surface terminations, we measured the differential conductance (or dI/dV) plots with STS on both the Pt_2_Te_2_ (Figure 1e, green curve) and the PtTe_2_ (Figure 1g, red curve) terminations, respectively. We find that the two different terminations show very different dI/dV characteristics over a wide range of the chemical potential (from −2.0 to 2.0 eV), highlighting distinct surface electronic properties and density of states (DOS) of the two terminations. Specifically, we observe a zero-bias peak in the local DOS for the PtTe_2_ (Figure 1g) surface termination, while such a feature is absent on the Pt_2_Te_2_-terminated surface (Figure 1e).

Below we will show explicitly that the zero-bias peak arises from an electron pocket in the surface states of the Pt_2_Te_2_-terminated surface.

### Bulk Band Structure and Topology

**Figure 2:**
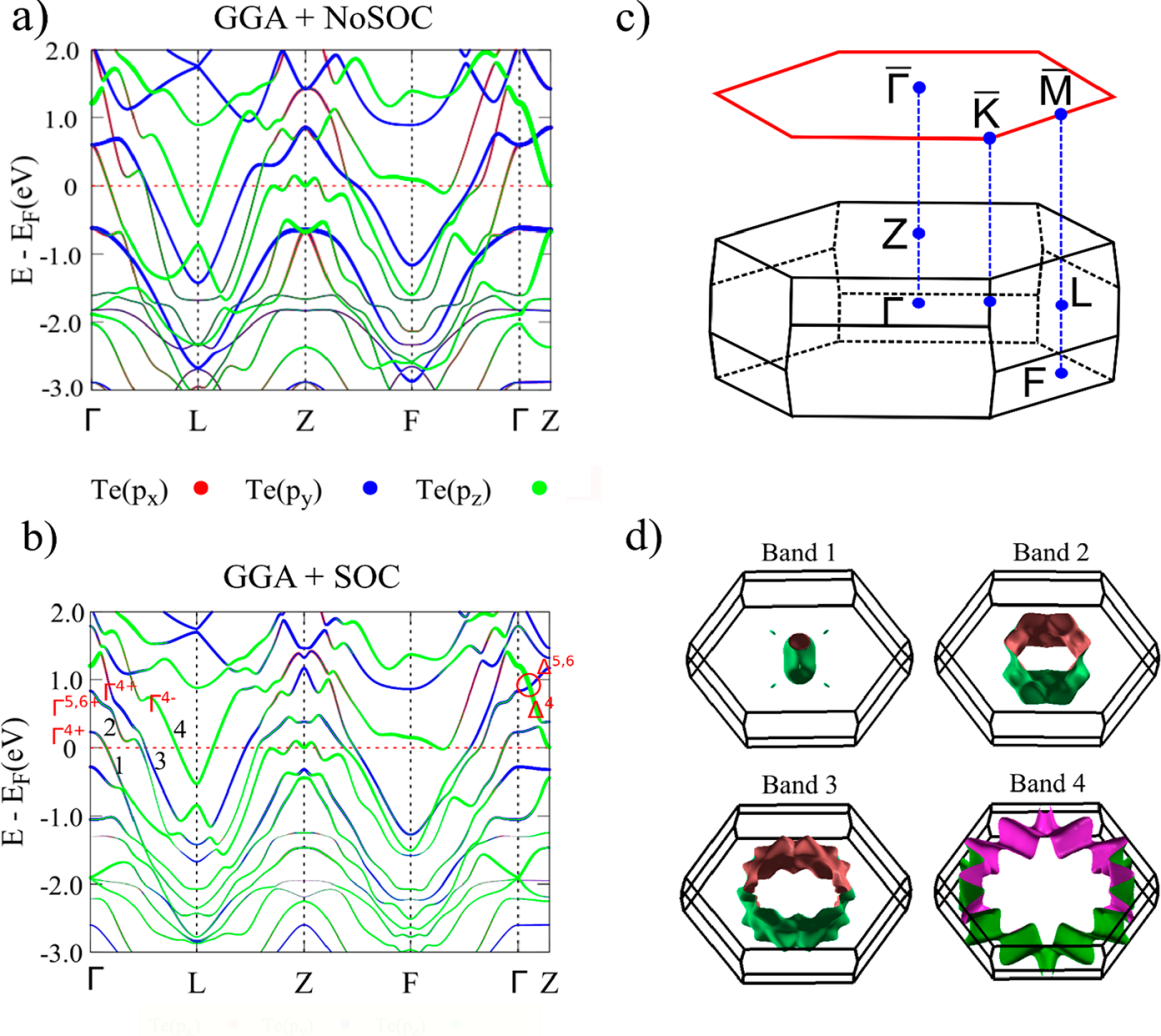
Calculated bulk band structure of Pt_3_Te_4_ along with the orbital character (a) excluding and (b) including the spin–orbit coupling. The irreducible representations of the bands at the Γ point are shown. The Dirac point along the Γ-Z direction is highlighted in the red circle. There are four bands crossing the Fermi level—marked as bands 1, 2, 3, and 4. Bands 1, 2, and 3 primarily result in hole pockets, while band 4 mainly leads to an electron pocket. (c) The bulk and the (111) surface projected Brillouin zone of the primitive cell with the high-symmetry points marked explicitly. (d) Fermi surfaces for four bands crossing the Fermi surface, clearly showing the presence of several hole and electron pockets arising from bands 1–4.

In order to further assess the dissimilarities in the surface electronic properties of the two terminations, we explored the electronic structure and spin texture of Pt_3_Te_4_. The bulk electronic band structure, without and with including the spin–orbit coupling (SOC) is shown in Figure 2a, b. The band structure clearly indicates the metallic character of the system. We find that the bands near the Fermi level predominantly arise from the Te *p* states. The interplay between the different chalcogen *p*-derived states gives rise to a type-I bulk Dirac Fermion along the Γ-Z direction of the Brillouin zone. This Dirac cone is also protected by the rotation symmetry, as confirmed by our symmetry analysis. The bands marked by Δ^5,6^ and Δ^4^ (see Figure 2b) have opposite rotation (C_3_) characters (−1 and +1, respectively) which prevents their hybridization and stabilizes the Dirac crossing. Other than this Dirac crossing, four different bands (marked explicitly in Figure 2b) cross the Fermi level, resulting in several hole and electron pockets. The Fermi surfaces corresponding to these four bands are shown in Figure 2d.

The bulk band structure of mitrofanovite displays multiple crossings between the valence and the conduction bands. This indicates the possibility of formation of topologically protected states. Furthermore, the Γ^4+^ (band 3) and the Γ^4–^ (band 4) bands are locally separated by a gap at every k-point. Therefore, to pin down the exact topology of this system, we computed the  topological invariants. As Pt_3_Te_4_ preserves the inversion symmetry, the topological invariant is calculated using the parity-based method developed by Fu-Kane.^25^ The details of the number of occupied bands with a specific parity is presented in Table S1 of the Supporting Information. Based on the calculated parity of the occupied bands, the strong topological invariant (ν_0_) is found to be zero and all the three weak topological invariants are nonzero. The four component  invariant is found to be (0; 111). As Pt_3_Te_4_ hosts Dirac cones, it has a Fermi surface like a metal, and it is characterized by a nontrivial topological index, which results in spin-polarized topological surface states, we classify it as a topological metal.

### Spin-ARPES Results

The nontrivial topological invariant indicates the presence of topological surface states, displaying spin-momentum locking. To experimentally explore the topological surface states and their spin texture, we used spin- and angle-resolved photoemission spectroscopy (spin-ARPES).

**Figure 3:**
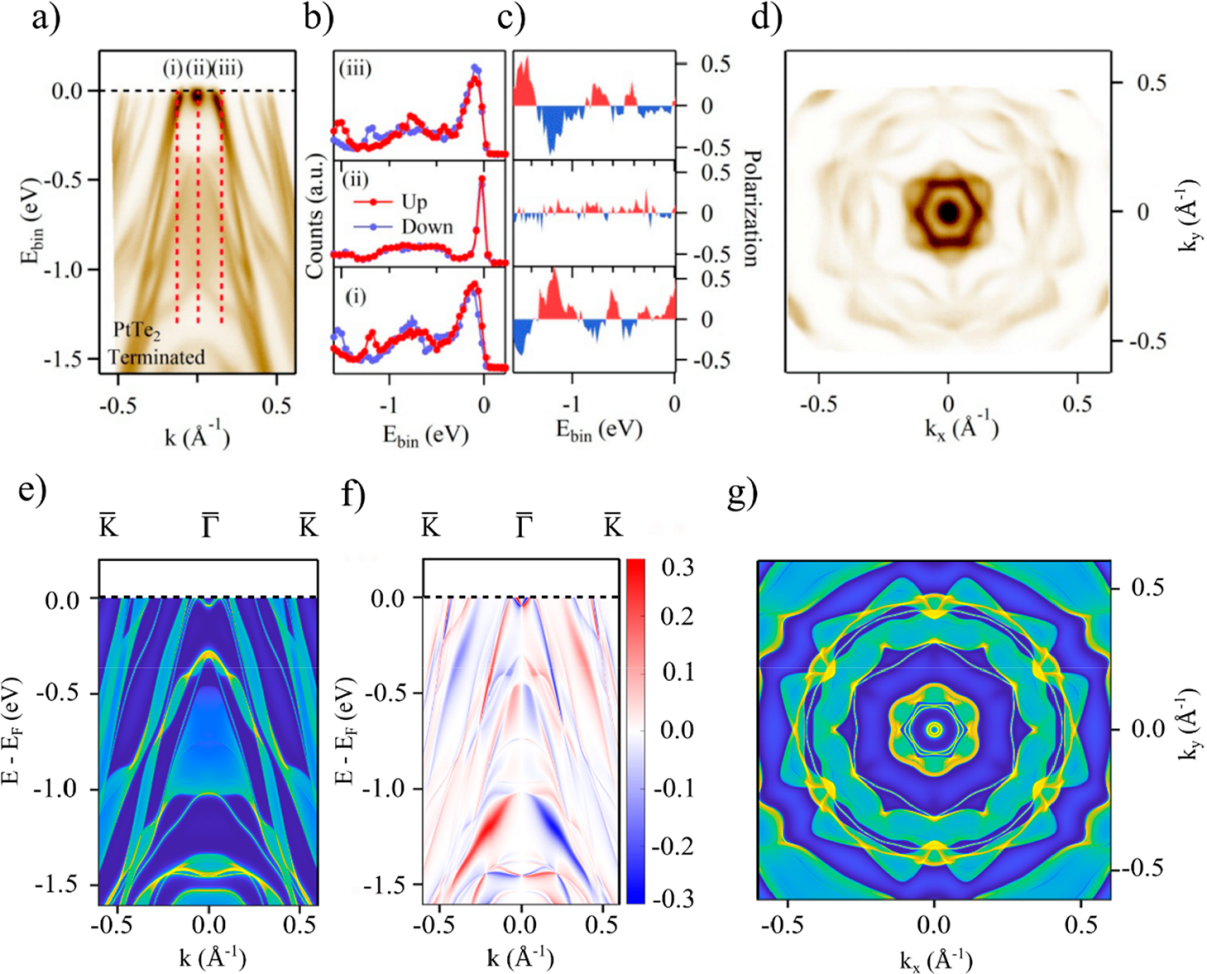
Experimental results and theoretical calculations for the PtTe_2_ termination. (a) Band dispersion along Γ-K measured with *h*ν = 22 eV. Red dotted lines indicate the positions in the momentum space where the spin spectra in part b and the resulting spin polarization in part c were measured. All spin data are related to the component perpendicular to the momentum (i.e., perpendicular to Γ–K), and there is a clear spin inversion between positive and negative momenta (i.e., between (i) and (iii)). (d) Measured Fermi surface.; (e) Calculated spectral function and (f) the corresponding spin texture. (g) Calculated Fermi surface.

**Figure 4:**
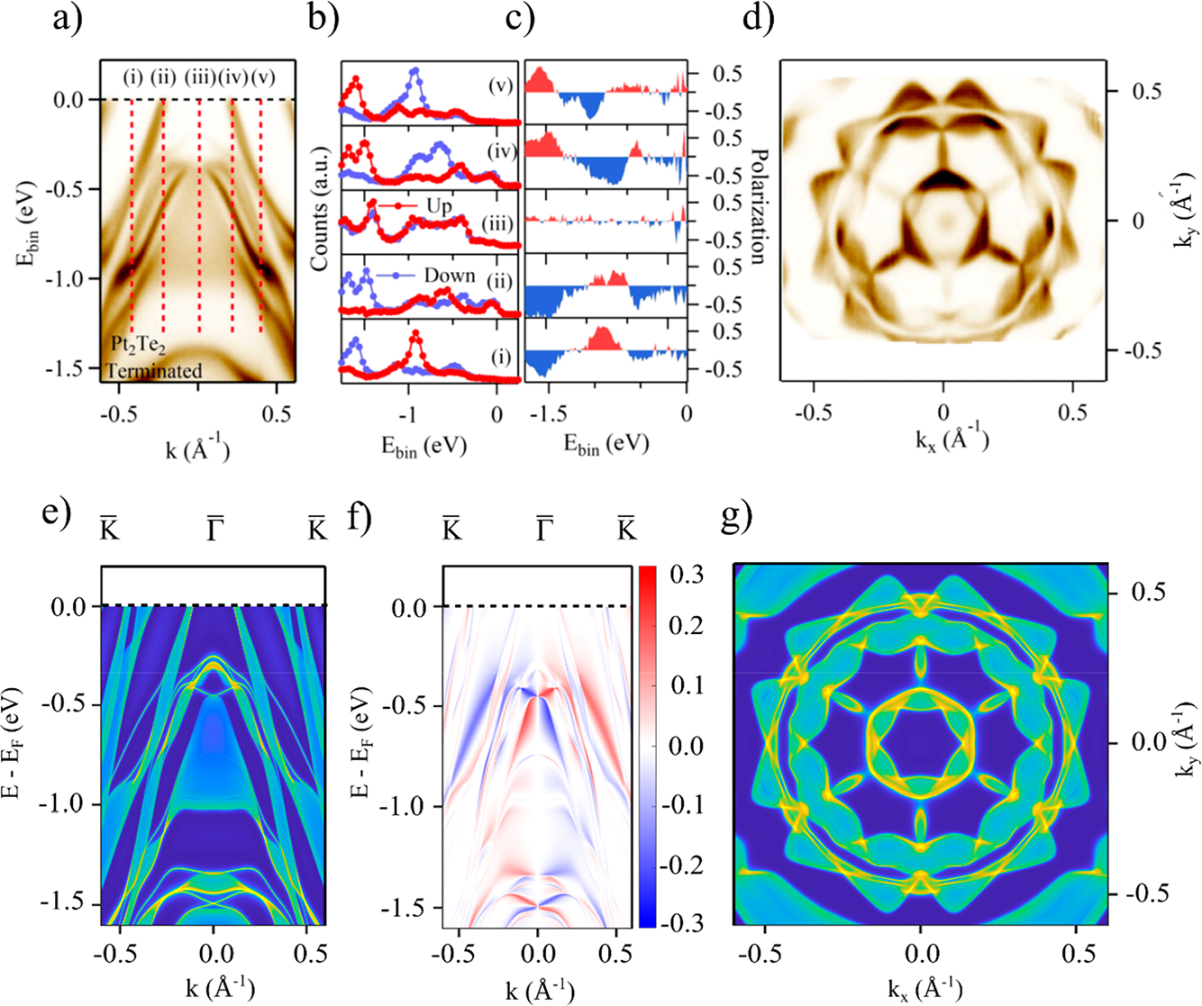
Pt_2_Te_2_ termination. Experimental results and theoretical calculations for the Pt_2_Te_2_ termination: (a) Band dispersion along the surface Γ-K direction measured with *h*ν = 22 eV; red dotted lines indicate the positions in the momentum space where the spin spectra in part b and the resulting spin polarization in part c were measured; all spin data are related to the component perpendicular to the momentum (i.e., perpendicular to Γ–K), and there is a clear spin inversion between positive and negative momenta. (d) Measured Fermi surface. (e) Calculated spectral function. (f) Corresponding spin texture. (g) Calculated Fermi surface.

Given the surface sensitivity of the technique (probing depth of the order of 10 Å in our experimental conditions), we find that the measured electronic band structure is strongly termination dependent. This is evident in the two distinct data sets reported in Figures 3 and 4 obtained on the same sample just by changing the position between the two different terraces. The measured electronic dispersion along the surface Γ-K direction, for the PtTe_2_ and the Pt_2_Te_2_ terminations, is shown in Figures 3a and 4a, respectively, with the corresponding Fermi surfaces reported in Figures 3d and 4d. We ascribed those data to the two terminations by comparing the experimental results with the theoretically calculated surface band structure for a semi-infinite geometry terminating in either the PtTe_2_ or the Pt_2_Te_2_.

For the PtTe_2_ termination, we find that the surface spectral function has several bands, including the surface termination-induced electron pocket at the center of the Brillouin zone (see Figure 3a and 3e). In addition, we also identify four hole-like bands crossing the Fermi energy. Since these spectral features show almost no dependence on the photon energy (i.e., no *k*_*z*_ dispersion is observed, see Supporting Information Figure S2), all of them can be ascribed to the two-dimensional surface states. The measured Fermi surface for the PtTe_2_ termination in Figure 3d clearly shows that the electron pocket at Γ is surrounded by a hole pocket with 6-fold rotation symmetry. The spin-polarization of these states is measured along three representative *k* points (Γ and two symmetric points at positive and negative *k*) marked by dashed lines in Figure 3a. We acquired data for the spin in-plane direction perpendicular to the crystal momentum, typically indicative of spin-momentum locking.

The resulting spin resolved spectra and spin polarization are shown in Figure 3b and 3c, respectively. While at Γ (Figure 3b) the spin up (red) and spin down (blue) spectra are the same, resulting in zero spin polarization (Figure 3c), the situation is different for the two spectra taken at the two symmetric *k* points around Γ. In fact, in panels (i) and (iii) the blue and red spectra are inverted, while the spin polarization is basically the same, but of opposite sign, in agreement with the spin-momentum locking. Note that not only the states at the Fermi energy exhibit spin polarization. It extends over the whole energy range probed in our experiment. All these features are well captured by our theoretical calculations for the semi-infinite geometry (see Figure 3e–g).

In contrast to the numerous bands crossing the Fermi energy for PtTe_2_, for Pt_2_Te_2_ termination only two evident Fermi crossings along the ΓK direction (Figure 4a) exist. In general, for the Pt_2_Te_2_ termination, the number of electronic states below the Fermi energy is relatively lower as compared to the PtTe_2_-terminated surface. Additionally, the surface electron pocket at the Γ point in the vicinity of the Fermi energy, observed for the PtTe_2_ termination, is absent for the Pt_2_Te_2_ termination (see Figure 4a). These differences are also reflected in the zero-bias peak observed on the PtTe_2_ but not on Pt_2_Te_2_ terrace, where the number of states at the Fermi energy is relatively low. The theoretical Fermi surface plots for the two terminations indicate that both of them display a 6-fold rotation symmetry (see Figures 3d and Figure 4d). Similar to PtTe_2_ termination, also on Pt_2_Te_2_ we detect spin-polarized states (Figure 4b and c) with the polarization inversion across Γ reflecting spin-momentum locking, evidenced as well in the calculated spin-resolved dispersion in Figure 4f.

Independent of the band structure differences, for both the terminations the calculated spin-resolved electronic spectral function (see Figures 3f and 4f) indicates that mitrofanovite is a strongly spin–orbit interacting system, with the surface states displaying spin-momentum locking. From the data in Figure 3b–c and in Figure 4b–c, for the PtTe_2_ and Pt_2_Te_2_ terminations, respectively, we find that for both the terminations the spin polarization is zero at the Γ point (Figure 3c, panel (ii), and Figure 4c, panel (iii). However, the spin polarization is finite and reaches up to 50% on the two sides of the Γ point, with clear polarization inversion of the corresponding states (between panels (i)–(iii) for PtTe_2_ and between panels *(*i)–(v) and (ii)–(iv) for the Pt_2_Te_2_ termination). Additionally, we observed spin-polarized states exhibiting spin-momentum locking over the full energy range that we probed. This observation is consistent with calculations for the spin-texture of the surface states in a finite-slab geometry (Figures 3f and 4f). The large value of the measured spin polarization and the spin-momentum locking are also consistent with the topological origin of the observed surface states and the topological nature of Pt_3_Te_4_.

Interestingly, Pt-class type-II Dirac semimetal PtTe_2_ also hosts multiple topological surface states in a wide energy range, which arise from various band inversions within the Te p-orbital manifold.^26,27^ Similarly to PtTe_2_, Pt_3_Te_4_ exhibits topological surface states deep in the valence bands. However, there are significant differences in the surface states. For example, the Dirac-like surface state at the Γ point observed for the PtTe_2_ termination in Pt_3_Te_4_ is absent in bulk PtTe_2_. Moreover, PtTe_2_ hosts an electron-pocket-like surface state near the Fermi level, which arises from the band inversions in the upper conduction bands, which are absent in Pt_3_Te_4_.

The observed surface states with robust spin polarization in Pt_3_Te_4_ can be exploited for making electrical devices for spin-injection and spin-detection.^28^ Furthermore, the spin-momentum locked states in Pt_3_Te_4_ indicate that it can have a large spin–orbit torque. Thus, it can be used as a candidate in spin-torque devices for applications in computation, logic, and memories.^28^

Finally, as validated by atomic force microscopy (AFM) experiments in Supporting Information Figure S4, mitrofanovite-based surfaces also provide outstanding ambient stability even for prolonged storage in air. The chemical reactivity of both terminations is practically the same, as indicated by calculations reported in Table S1 of the Supporting Information.

## Conclusions

In summary, we have demonstrated that the recently discovered ambient-stable mitrofanovite Pt_3_Te_4_ is a topological metal with termination-dependent surface states and spin polarization using a combination of ARPES and STS experiments and *ab initio* calculations. More interestingly, we find that the two distinct terminations (PtTe_2_ and Pt_2_Te_2_) can arise as different terraces on the same face of the cleaved crystal. The two terminations have dissimilar electronic surface states, even though both terminations host spin-polarized surface states. The spin-polarized surface states display polarization reversal across the zone center, which is a characteristic feature of the spin-momentum locking. Our demonstration of termination-dependent electronic surface properties and spin polarization in Pt_3_Te_4_, combined with its excellent ambient stability, makes it an interesting candidate for exploring potential nanoelectronics, spintronics, optoelectronics, and plasmonic applications in mitrofanovite-based heterostructures and interfaces.

## Methods

### Single-Crystal Growth

Single crystals of Pt_3_Te_4_ were grown by the self-flux method. Unlike the case of PtTe_2_,^29^ the growth window of Pt_3_Te_4_ is narrow. The mixtures of high-purity Pt foil and Te ingots with the molar ratio of 51:49 were inserted in an alumina crucible and sealed into an evacuated quartz ampule. The quartz ampule was heated to 1080 °C for 24 h and then slowly cooled to 975 °C at a rate of 1 °C/h. The excess flux was separated by centrifugation above 970 °C and mechanical polishing. Shiny platelike Pt_3_Te_4_ single crystals were harvested with a dimension of 4 × 3 × 0.4 mm^3^. The flat surface of the crystal corresponds to the (001) plane, as identified by XRD analyses reported in Figure S1 of the Supporting Information.

### STM-ARPES

STM/STS were obtained with in situ STM/S (introduced in ref (30)) on the same surfaces for which the ARPES data in Figures 3 and 4 were collected. The topographic images (Figure 1e and the right-side insets) were measured with constant current mode. The dI/dV curves (Figures 1f and 1h) were numerically obtained from the measured I–V curves. All STM/STS experiments were carried out at room temperature.

Spin-ARPES experiments were carried out on the APE-LE beamline at Elettra synchrotron, described in ref (31). We note here that with the available beam-spot size on the sample of 150 × 50 μm^2^, we could clearly distinguish between two different situations regarding the electronic band structures (as summarized in Figures 3 and 4) simply by moving the sample in front of the beam. This is feasible, considering that the size of terraces on as-cleaved Pt_3_Te_4_ surfaces exceeds 150 μm^2^.

### Computational Framework

First-principles calculations for the electronic properties were carried out using DFT, as implemented in the Vienna *ab initio* Simulation Package (VASP).^32,33^ The exchange-correlation effects were incorporated using the Perdew–Burke–Ernzerhof (PBE) implementation of the generalized gradient approximation (GGA).^34^ A plane wave basis set with a cutoff energy of 500 eV was used. The ionic relaxation was performed until the force on each atom was lower than 10^–3^ eV/Å. The self-consistency convergence criteria for the total energies were set to 10^–7^ eV, and a Γ-centered 8 × 8 × 8 Monkhorst–Pack *k*-point grid^35^ was used to perform the Brillouin zone integration. We constructed the tight binding model with the atom centered “Wannier-like” orbitals using the VASP2WANNIER90^36^ interface. A surface energy spectrum was obtained within the iterative Green’s function method following the implementation in the WannierTools package.^37^

## Supporting Information Available

Additional information about (i) experimental characterization, (ii) the topological invariants, (iii) ARPES measurements, and (iv) ambient stability of Pt_3_Te_4_ (PDF)
