# Supplementary material for: Mitrofanovite Pt3Te4: A Topological Metal with Termination-Dependent Surface Band Structure and Strong Spin Polarization
Source: ACS Nano. 2021 Sep 2;15(9):14786–93. doi: 10.1021/acsnano.1c04766 (PMC8482756; doi:10.1021/acsnano.1c04766)
Supplement: Supplementary file 1 — nn1c04766_si_001.pdf [file nn1c04766_si_001.pdf]

Supporting Information for the paper

Mitrofanovite Pt_3Te_4: a Topological Metal with Termination-Dependent Surface Band Structure and Strong Spin Polarization

Jun Fujii [1,§], Barun Ghosh [2,§], Ivana Vobornik [1,§, *], Anan Bari Sarkar[2], Debashis Mondal[1], Chia-Nung Kuo[3], François C. Bocquet[4,5], Lixue Zhang[6], Danil W. Boukhvalov[7,8], Chin Shan Lue[3], Amit Agarwal[2,*], Antonio Politano[9,10,*]

[1] CNR-IOM, TASC Laboratory, Area Science Park-Basovizza, 34139 Trieste, Italy
[2] Department of Physics, Indian Institute of Technology Kanpur, Kanpur, 208016, India
[3] Department of Physics, National Cheng Kung University, 1 Ta-Hsueh Road, 70101 Tainan, Taiwan
[4] Peter Grünberg Institut (PGI-3), Forschungszentrum Jülich, 52425 Jülich, Germany
[5] Jülich Aachen Research Alliance (JARA), Fundamentals of Future Information Technology, 52425 Jülich, Germany
[6] College of Science, Institute of Materials Physics and Chemistry, Nanjing Forestry University, Nanjing 210037, P. R. China
[7] College of Science, Institute of Materials Physics and Chemistry, Nanjing Forestry University, Nanjing 210037, P. R. China
[8] Institute of Physics and Technology, Ural Federal University, Mira Street 19, 620002 Ekaterinburg, Russia
[9] Department of Physical and Chemical Sciences, University of L'Aquila, via Vetoio, 67100 L'Aquila (AQ), Italy
[10] CNR-IMM Istituto per la Microelettronica e Microsistemi, VIII strada 5, I-95121 Catania, Italy

Synchrotron X-ray powder diffraction

The in-house XRD system is insufficient for phase identification and structure analysis due to the malleability of Pt_3Te_4 crystals. To reduce the broadening of peaks and preferential orientation, a synchrotron x-ray powder diffraction (SXRD) experiment was conducted. The SXRD patterns were collected from 100 to 480 K with the MYTHEN detector with 15 keV beam at beam line 09A, Taiwan Photon Source, National Synchrotron Radiation Research Center (NSRRC) in Hsinchu, Taiwan. The single crystal was pulverized and packed in a 0.1 mm borosilicate capillary to minimize the absorption effect. The capillary was kept spinning during data collection for powder averaging.

As shown in Fig. S1a, all diffraction peaks match well with the Pt_3Te_4 structure (ICSD # 41372). Fig. S4b shows the SXRD patterns in the temperature range 100 - 480 K. The absence of extra reflections also confirmed the structural stability of Pt_3Te_4.

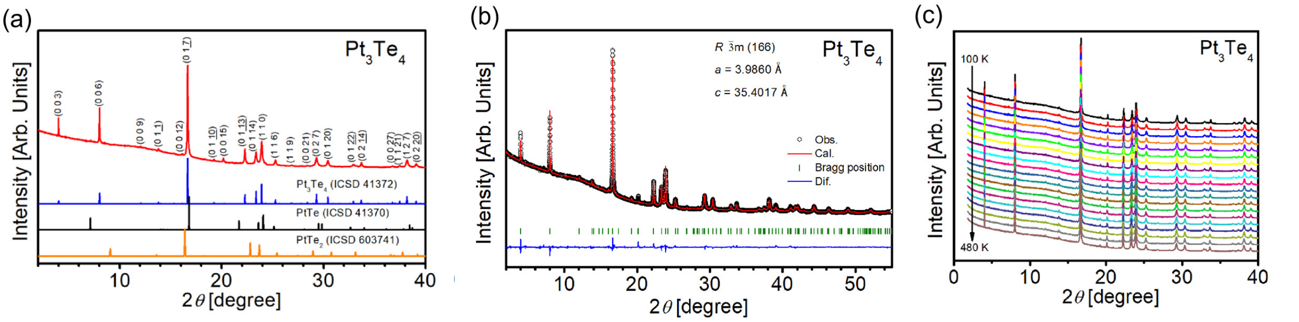

Figure S1. (a) The SXRD of the Pt_3Te_4 crystal at T=300 K. The simulated SXRD patterns of Pt_3Te_4, PtTe, and $PtTe_2$ are shown below for the sake of comparison. (b) The Rietveld refinement of the SXRD of Pt_3Te_4 crystal at T=300 K. (c) Selected SXRD patterns of Pt_3Te_4 in the temperature range of 100 – 480 K with an interval of 20 K.

$\mathbb{Z}_2$ topological invariants

Based on the parity data of the occupied bands, the strong topological invariant (ν_0) is found to be zero and all the weak topological invariants are non-zero. The exact $_2$ invariant is found to be (0; 111). Therefore, from the standpoint of the topology of the electronic band structure, Pt_3Te_4 is a weak topological metal.

Table S1. The number of occupied bands of positive (n_{occ}^+) and negative parity (n_{occ}^-) eigenvalues at eight TRIM points.

**Γ**	(0,0,0)	(π,0,0)	(0,π,0)	(0,0,π)	(π,π,0)	(π,0,π)	(0,π,π)	(π,π,π)
n_{occ}^+	21	20	20	20	20	20	20	19
n_{occ}^-	18	19	19	19	19	19	19	20

Photon-energy dependent ARPES spectra

Figures S2 and S3 report photon-energy dependent ARPES spectra for $PtTe_2$- and Pt_2Te_2-terminated Pt_3Te_4 surfaces, respectively.

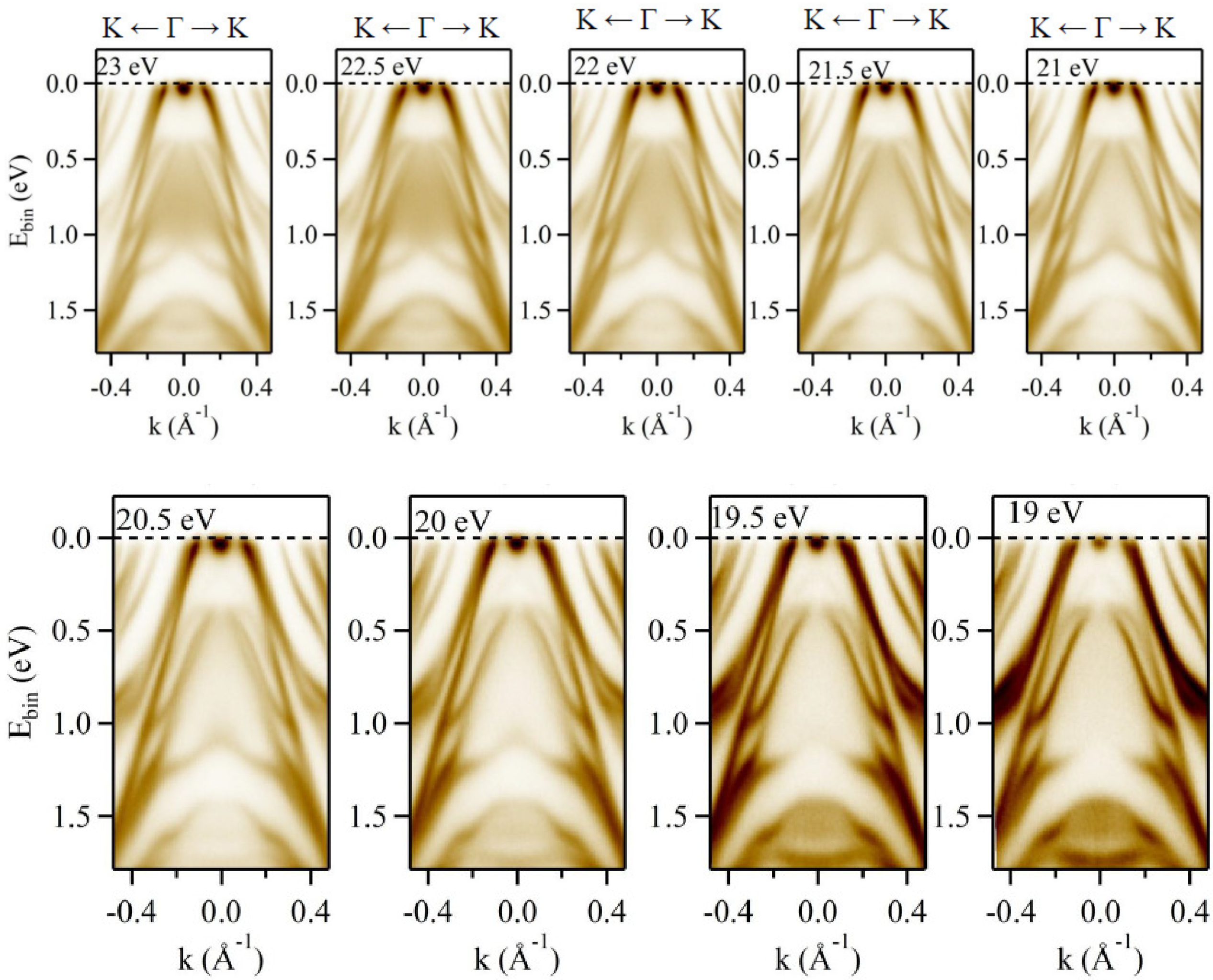

Figure S2. Photon-energy dependent ARPES spectra for the $PtTe_2$-terminated Pt_3Te_4 surface.

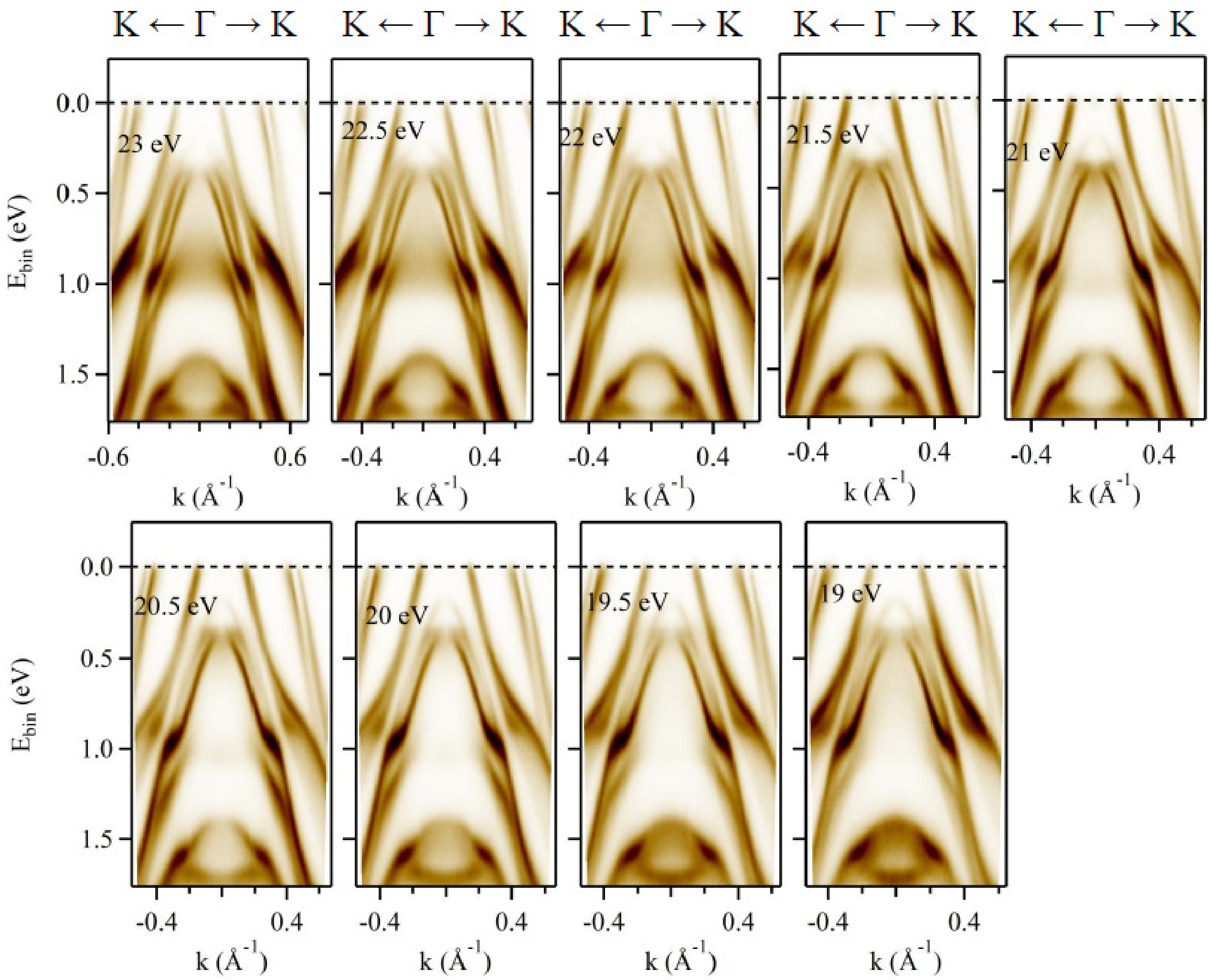

Figure S3. Photon-energy dependent ARPES spectra for the Pt_2Te_2-terminated Pt_3Te_4 surface.

Ambient stability

The ambient stability was also assessed by a morphological investigation by atomic force microscopy (AFM) in a timescale extended up to one month. The AFM experiments demonstrate that exposure to

air did not modify the morphology of the Pt_3Te_4 surface (Fig. S4), as established by the minimal changes in the height profile along a specific direction.

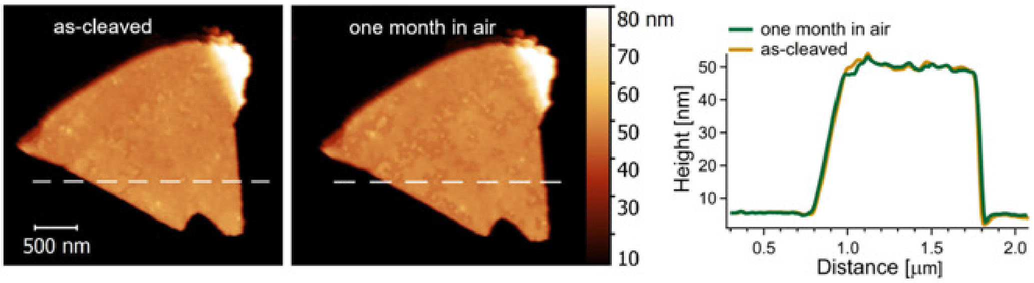

Figure S4. Morphological evolution of a Pt_3Te_4 flake of 70 nm from (left panel) sample preparation up to (middle panel) a prolonged exposure to air (one month). (right panel) Height profile as a function of the distance along dashed white lines in panels (left) and (middle).

To reveal the chemisorbed species upon air exposure, we carried out high-resolution electron energy loss spectroscopy (HREELS) experiments. This technique has inherently the required chemical specificity requested for addressing referee's point.

The analysis of the vibrational spectrum clearly indicates the emergence of CHx species upon air exposure. As a matter of fact, the vibrational spectrum exhibited the various infrared-active modes of CHx species from airborne contamination: (i) wagging at 1152 cm^{-1}; (ii) rocking at 1305 cm^{-1}; (iii) scissoring at 1485 cm^{-1}; (iv) stretching at 2964 cm^{-1}, respectively. On the other hand, TeO_2 is missing.

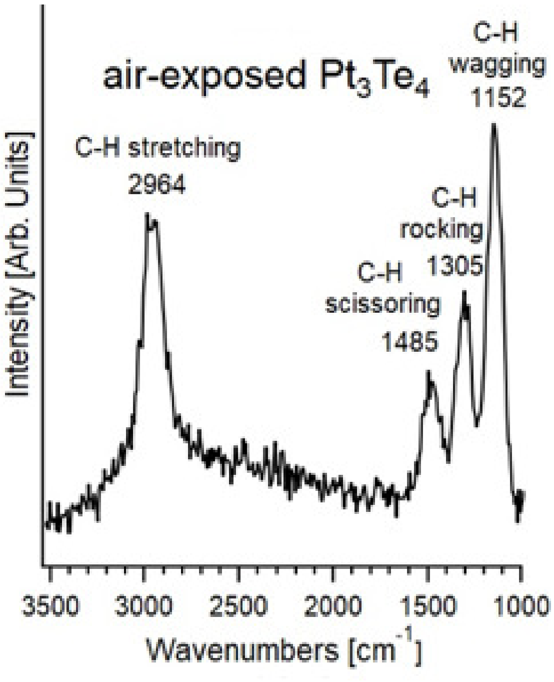

Figure S5: Vibrational spectrum of air-exposed Pt_3Te_4

We also assessed oxidation robustness of the two surface terminations by density functional theory. Results of the calculations (Tab. S1) demonstrates favorability of physical adsorption of molecular oxygen on both types of surfaces. Further decomposition of the *single* oxygen molecule is also exothermic process. Note that decomposition of molecular oxygen requires activation of the oxygen molecule. Activation energy is usually considered as the energy required for triplet-singlet transition of O_2 molecule (~120 kJ/mol). Since the magnitude of the energy cost of activation larger than energy gain from decomposition (especially for $PtTe_2$ surface) we can discuss decomposition of molecular oxygen as rare events. Further oxidation of Pt_2Te_2 surface corresponds to the decreasing of the magnitude of the enthalpy, and oxidation of whole $PtTe_2$ surface is endothermic process. Additionally, this possible oxidation of the surface corresponds to the formation of Te-O bonds without further

oxidation of Pt and other Te-layers in surface layer. Thus, we can consider oxidation of any Pt_3Te_4 surface as spot-like self-limiting slow process.

Table S1. Differential enthalpy ΔH_{ads} *and differential Gibbs free energy* ΔG_{ads} *for physical adsorption and differential enthalpy of decomposition* ΔH_{dec} *(all in kJ/mol) for molecular oxygen on defect-free (i)* $PtTe_2$*- and (ii)* Pt_2Te_2*-terminated* Pt_3Te_4 *surfaces We also report the differential enthalpy of the oxidation of whole surface (in parenthesis).*

Surface termination of Pt_3Te_4	ΔH_{ads} [kJ/mol]	ΔG_{ads} [kJ/mol]	ΔH_{dec} [kJ/mol]
$PtTe_2$	-42.62	-31.13	-51.78 (+1.31)
Pt_2Te_2	-40.81	-29.51	-98.08 (-27.06)
